# Towards precision medicine-based therapies for glioblastoma: interrogating human disease genomics and mouse phenotypes

**DOI:** 10.1186/s12864-016-2908-7

**Published:** 2016-08-22

**Authors:** Yang Chen, Zhen Gao, Bingcheng Wang, Rong Xu

**Affiliations:** 1 Department of Epidemiology and Biostatistics, Case Western Reserve University, Cleveland, Ohio USA; 2 Department of Pharmacology, Case Western Reserve University, Cleveland, Ohio USA

**Keywords:** Glioblastoma, Drug repositioning, Cancer genomics, Mouse phenotype

## Abstract

**Background:**

Glioblastoma (GBM) is the most common and aggressive brain tumors. It has poor prognosis even with optimal radio- and chemo-therapies. Since GBM is highly heterogeneous, drugs that target on specific molecular profiles of individual tumors may achieve maximized efficacy. Currently, the Cancer Genome Atlas (TCGA) projects have identified hundreds of GBM-associated genes. We develop a drug repositioning approach combining disease genomics and mouse phenotype data towards predicting targeted therapies for GBM.

**Methods:**

We first identified disease specific mouse phenotypes using the most recently discovered GBM genes. Then we systematically searched all FDA-approved drugs for candidates that share similar mouse phenotype profiles with GBM. We evaluated the ranks for approved and novel GBM drugs, and compared with an existing approach, which also use the mouse phenotype data but not the disease genomics data.

**Results:**

We achieved significantly higher ranks for the approved and novel GBM drugs than the earlier approach. For all positive examples of GBM drugs, we achieved a median rank of 9.2 45.6 of the top predictions have been demonstrated effective in inhibiting the growth of human GBM cells.

**Conclusion:**

We developed a computational drug repositioning approach based on both genomic and phenotypic data. Our approach prioritized existing GBM drugs and outperformed a recent approach. Overall, our approach shows potential in discovering new targeted therapies for GBM.

## Background

Glioblastoma (GBM) is one of the leading causes of cancer-related deaths in both the pediatric and adult populations [1]. The standard treatment includes radiation plus chemotherapy following maximal safe resection of cancer mass [2]. However, the prognosis of GBM patients remains poor even with optimal radio- and chemo-therapies: the mean survival is 15 months and most patients die within two years [2, 3]. In addition, GBM is not a priority for new drug development because of socioeconomic problems and medical difficulties [3]. Both the grim prognosis and urgent clinical needs have motivated us to develop an “in silico” drug repositioning approach and pursue FDA-approved agents that has the potential to treat GBM but not previously identified as GBM therapeutics.

Since GBMs are highly heterogeneous at the genomic, histological and differentiation level, the lack of specific therapies contributes to the treatment failures. Cancer therapies that target on specific molecular profiles of individual tumors have the potential to maximize the efficacy [4]. For example, Imatinib has been used to successfully treat a subtype of leukemia with mutations in the BCR-ABL fusion protein and has achieved a median survival of five years [5]. Over the past two decades, extensive researches have identified hundreds of genetic mutations that likely drive the GBM formation [6, 7]. More recently, systemic multi-platform analysis of glioma and bioinformatic mining by The Cancer Genome Atlas (TCGA) has led to the classification of GBM into distinct molecular subtypes according to the genes altered during gliomagenesis [8, 9]. Here, we use the accumulated genomic data for GBM to guide the drug repositioning approach towards discovering precise targeted drugs for GBM.

**Fig. 1:**
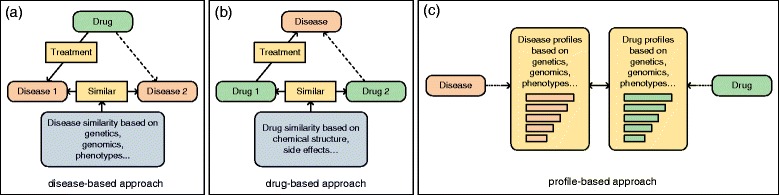
Computational drug repositioning strategies: **a** Disease-based methods (Similar diseases may be treated with the same drug), **b** Drug-based methods (similar drugs may treat the same disease), and **c** Profile-based methods (the association between a drug and a disease is estimated by their profile similarity)

Disease genetic and genomic profiles have been demonstrated useful in computational drug discovery approaches [10–15]. These approaches estimate the association between a drug and a disease through calculating their genomic profile similarities. They show increased ability in discovering new drug-disease pairs comparing with drug-based and disease-based repositioning strategies (Fig. 1), which depend on existing drug-indication knowledge to infer new drug-disease associations. However, the profile-based approach (Fig. 1(c)) has an inherent challenge: the lower-level genomics profile similarities between drugs and diseases do not necessarily translate into higher-level drug treatment efficacy in diseases. Previous studies have demonstrated that phenotypic data are critical in computational drug discovery approaches [16–19]. Recently, the Mouse Genome Informatics (MGI) database [20] has provided large amounts of phenotypic descriptions for mouse genetic mutations based on systematic gene knockouts, which are impossible on human. These causal gene-phenotype associations in mice have been demonstrated useful in discovering of new disease-associated genes [21] and drug targets [22], and also have the potential to overcome the challenge in genomics-based drug repositioning approaches.

In this study, we develop a novel GBM drug repositioning strategy leveraging both lower-level disease and drug genomics and higher-level mouse phenotypes. We first identify GBM-specific mouse phenotypes using a compiled list of GBM-associated genes identified by multiple TCGA studies [6, 8]. Then we screen all the FDA-approved drugs for candidates that share similar mouse phenotype profiles with GBM. We validate the approach using approved GBM drugs, and approximate the performance in detecting novel GBM drugs using two evaluation sets: a set of potential GBM therapies tested in clinical trials and a set of off-label GBM drugs in the post-marketing surveillance system. Finally, we investigate the top 10 % drug predictions. Overall, we combine the genomic and phenotypic data for diseases and drugs towards identifying novel targeted therapies for GBM.

## Methods

**Fig. 2:**
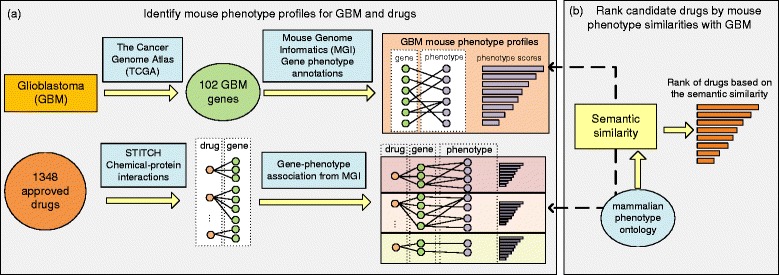
Our method contains two parts: **a** Identify mouse phenotype profiles for GBM and all approved drugs, and **b** Rank candidate drugs by mouse phenotype similarities with GBM

Our approach ranks 1348 approved drugs by the mouse phenotype profile similarities between GBM and each drug. Figure 2 shows two steps in the algorithm: (1) identifying the phenotypes in mice for GBM and each approved drug, using the well-studied disease-associated genes and drug target genes, respectively; and (2) calculating the semantic similarities of the mouse phenotype profiles between the disease and drugs. The rank of drugs based on the phenotype similarities suggests how likely the drug can be used to treat GBM. The following parts describe each step as well as the evaluation methods in detail.

### Identify mouse phenotype profiles for GBM and drugs using disease genetics and drug target genes

TCGA Research Network provides a comprehensive catalog of genomic abnormalities driving tumorgenesis. We compiled a list of 102 genetic mutations that significantly differentiate GBM tumors and healthy tissue from several recent TCGA studies [6, 8]. The list primarily contains the genes in the core GBM-associated pathways, including p53, Rb, and receptor tyrosine kinase (RTK)/Ras/phosphoinositide 3-kinase (PI3K) signaling.

We extracted the mouse phenotypes that are linked to 102 GBM-associated genes from the MGI database. Each phenotype was weighted and ranked by the number of genes it is linked with. The mouse phenotype terms were removed from the list if their weights are smaller than the median of all weights. At last, we identified a list of 945 GBM-specific mouse phenotypes. We mapped the phenotype terms into 26 categories by tracing the *isa* relationship in the mammalian phenotype ontology. A score was calculated for each category as the sum of weights of all phenotypes in it. We ranked the phenotype categories by their scores and investigated the top five categories.

Then we identified the mouse phenotype profile for each of the 1348 FDA-approved drug. The drug target genes were first extracted from the STITCH database, and each drug-target link has a confidence score. Then we extracted the mouse phenotypes that are linked with the target genes for each drug. The phenotype terms are weighted by the sum of confidence scores of the corresponding target genes. Finally, we obtained a vector of weighted mouse phenotype features for each candidate drug.

### Rank candidate drugs for GBM using mouse phenotype similarities between GBM and drugs

We calculated the phenotypic similarity between GBM and the drugs in order to rank the candidate drugs by their similarity to GBM. Phenotype terms associated with both GBM and the drugs were normalized by concepts in the ontology, which provides semantic relationships between concepts and has been widely used in biomedical applications [17, 21, 23, 24]. We calculated the semantic distances between the mouse phenotype vectors for GBM and the candidate drugs in the context of the mouse phenotype ontology.

We first quantified the information content for each phenotype term *t* as −*logp*(*t*), in which *p*(*t*) represents the frequency among phenotype annotations to all the 7568 mouse genes. In calculating the information content, if a gene is annotated by one phenotype term, we assumed that it is also annotated by the ancestors of this term in the hierarchy of mammalian phenotype ontology. Hence, a phenotype term has higher information content than its ancestors, which lie on higher levels in the ontology.

Then we defined the semantic distance *sim*(*t*_1_,*t*_2_) between phenotype terms *t*_1_ and *t*_2_ as:

1 $$ {\text{sim}}({t_{1}},{t_{2}}) = \mathop{\max}\limits_{a \in A({t_{1}},{t_{2}})} - \log p(a),  $$

where *A*(*t*_1_,*t*_2_) is the set of common ancestors for *t*_1_ and *t*_2_ in the ontology. To calculate the distance from the phenotype vector *p*_1_ to *p*_2_, we matched each phenotype feature in *p*_1_ to the most similar feature in *p*_2_ and took the average:

2 $$ {\text{sim}}({p_{1}} \to {p_{2}}) = avg \left[\sum\limits_{{t_{1}} \in {p_{1}}} {\mathop{\max}\limits_{{t_{2}} \in {p_{2}}}}\qquad{sim({t_{1}},{t_{2}})}\right]  $$

To calculate the distance between *p*_1_ and *p*_2_, we averaged the semantic distances in both directions:

3 $$ {\text{sim}}({p_{1}},{p_{2}}) = \frac{1}{2}{\text{sim}}({p_{1}} \to {p_{2}}) + \frac{1}{2}{\text{sim}}({p_{2}} \to {p_{1}})  $$

A similar definition of distance between a pair of concepts in an ontology was also used before [24].

### Validate our approach through de novo prediction of approved, potential, as well as off-label GBM drugs

We tested whether our approach can prioritize the existing and novel GBM drug therapies in the top among 1348 candidates. We compiled three evaluation drug sets based on previous studies [25, 26]: the approved GBM drugs, potential GBM drugs that have been tested in clinical trials, and off-label GBM drugs identified from a post-marketing drug surveillance system. The approved GBM drug set contains temozolomide and carmustine, which are cytotoxic (non-targeted) chemical drugs, and bevacizumab, which is the first targeted drug approved for brain tumor. The potential GBM drug set contains 52 drugs collected from the clinical trials. In addition, the FDA drug surveillance system contains large-scale drug-disease data collected from hospitals, patients, and pharmaceutical companies. A total of 36 off-label uses for GBM were extracted from this system (containing zero overlap with the 52 potential GBM drugs). We have removed the approved GBM drugs from both the potential and off-label GBM drug sets, and evaluated the ranks for these two sets to approximate the performance of the proposed approach in predicting novel GBM drugs.

We compared the performance of our approach with a recent drug repositioning approach proposed by Hoehndorf [27] in ranking the above evaluation sets. The Hoehndorf’s method also used the mouse phenotype data, but did not incorporate the human disease genomics data. They matched the human phenotype ontology [24] and the mammalian phenotype ontology [28] to predict genes for a human disease using the gene-phenotype relationships in animal models. After that, they linked the predicted disease genes with the drug target genes to suggest candidate drugs for the given disease.

We first evaluated the ranks for approved GBM drugs, and the median ranks for the potential and off-label GBM drug sets. We tested the median rank instead of the average, because the median is not affected by individual large ranks. For example, if the rank for a GBM drug is 1348 using method A and 1000 using method B, both methods fail in detecting this positive example. But method B may achieve much higher average ranks than method A, affected by the large values of these two ranks. We also extracted the overlapping drugs between the ranked drug lists generated by the two methods, and performed the paired student’s t-test to evaluate the significance of their ranking difference. Then we combined the three evaluation sets, assumed all the drugs as the positive examples, and compared the precision-recall curve as well as the mean average precision between methods.

## Result

### Identified mouse phenotypes are associated with GBM pathogenesis

We classified the GBM-specific mouse phenotypes detected through GBM-associated genes, and ranked the phenotype categories. Table 1 shows that the top-ranked phenotype categories are “tumorigenesis” and “nervous system phenotype” as expected. Besides, the result shows that GBM interacts with the immune system and hematopoietic system, which is consistent with a series of previous researches. A recent mouse model study [29] reveals that the GBM cells are able to migrate along the cerebral blood vessels and extract nutrients from the blood for themselves. They also replace the specialized brain cell named astrocytes to create a breakdown in the blood-brain barrier (BBB), which tightly controls the lymphocyte traffic into the central nervous system (CNS) in healthy people. Then the GBM cells evade the immune responses through inhibiting the T cell proliferation [30], inducing immunosuppressive microglia [31] and other channels. Studies on the pathways involving these immune evasion strategies have led to several recent advances in developing targeted immunotherapies for GBM [32, 33].

**Table 1:** The top-ranked categories of GBM-specific mouse phenotypes detected through disease genetics

Rank	Phenotype category	Example phenotype
1	Tumorigenesis	Increased glioblastoma incidence
2	Nervous system phenotype	Abnormal astrocyte morphology
3	Hematopoietic system	Abnormal hematopoiesis
	Phenotype	
4	Mortality/aging	Decreased survivor rate
5	Immune system phenotype	Decreased leukocyte cell number

### Our approach outperforms an existing drug repositioning approach in prioritizing approved, potential and off-label GBM drugs

Using the phenotype profiles detected through GBM and drug genomics, our approach prioritized the approved GBM drug bevacizumab in top 24.4 % among a total of 1348 chemicals, which is a much higher rank than Hoehndorf’s rank in 67.9 %. In addition, we identified the other two GBM approved drugs temozolomide and carmustine and ranked them within top 6.7 % and 10.7 %, respectively, while Hoehndorf’s ranking list does not contain these two drugs.

Table 2 shows that our median rank for the potential GBM drugs in clinical trials is 7.8 %, which is 4.8-fold higher than Hoehndorf’s median rank. For the off-label GBM drugs from the post-marketing surveillance system, our median rank is 15.3 %, which is 1.8-fold higher than Hoehndorf’s approach. We generated significantly better ranks for both the potential drug set (*p*=0.003) and the off-label drug set (*p*=0.02) than Hoehndorf’s approach based on result of the paired t-test. Besides, we ranked the potential drugs higher than the off-label drugs, possibly because of the mixed and noisy data sources of the post-marketing drug surveillance system.

**Table 2:** The ranks for GBM drugs in the three evaluation sets generated by our approach and Hoehndorf’s approach

Evaluation drug (set)		Our approach	Hoehndorf’s approach	*p*-value
Approved drugs	Temozolomide	6.7 %	NA	NA
	Carmustine	10.7 %	NA	NA
	Bevacizumab	24.4 %	67.9 %	NA
Potential drugs (clinical trials)		7.8 %	45.4 %	*p*=0.003
Off-label drugs (post-marketing surveillance)		15.3 %	44.2 %	*p*=0.02
Combination		9.2 %	45.6 %	*p*=0.0003

**Fig. 3:**
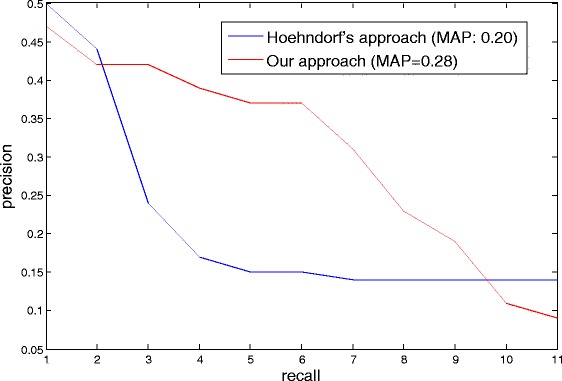
Precision-recall curve in ranking the positive examples of GBM drugs for our approach and Hoehndorf’s approach

We combined the three evaluation sets as a positive sample set and found their median rank is within top 9.2 % (Table 2). Comparing with Hoehndorf’s approach, we achieved significantly better performance in ranking these positive drugs (*p*=3*e*^−4^). Figure 3 shows the precision-recall curve for the two methods. The mean average precision calculated based on the curve is 0.29 for our approach, comparing to 0.20 for Hoehndorf’s approach.

We classified the drugs in all evaluation sets into three types, namely non-targeted cancer drugs, targeted cancer drugs and non-cancer drugs (Table 3). Our approach achieved the best performance in ranking the targeted cancer drugs, which has a median rank of 7.3 %. On the other hand, Hoehndorf’s approach performed best when predicting the non-targeted cancer therapies. This may be due to the different input data for the two methods: we incorporated the disease genomics data, while Hoehndorf’s approach directly analyzed the disease phenotypes. Overall, our approach works better than the baseline approach in ranking the evaluation drugs, which are more likely to be able to treat GBM than random drugs. The most significant difference between our approach and the baseline approach lies in ranking the non-cancer drugs that have been tested or in off-label use for GBM, and the paired t-test yielded a *p*-value of 6*e*^−4^.

**Table 3:** Median ranks for different types of drugs in the combined evaluation set

Drug type	Our approach	Hoehndorf’s approach	*p*-value
Non-targeted cancer therapies (chemotherapies)	9.5 %	25.8 %	*p*=0.023
Targeted cancer drugs	7.3 %	56.4 %	*p*=0.015
Non-cancer drugs	13.3 %	67.4 %	*p*=0.0006

Together, the result suggests that our approach performed significantly better than an existing method that also utilizes the mouse phenotype data in prioritizing all approved and novel GBM drugs, and specially in identifying potential targeted GBM drugs. One possible reason is that we used the most recent discoveries of GBM associated gene mutations and a more comprehensive drug-target database, which provides opportunities for discovering targeted therapies for GBM.

Table 4 lists five examples in our top 5 % predictions and their traditionally approved indications. Among them, rosiglitazone is a PPAR *γ* agonist that shows the ability to inhibit proliferation of human GBM cell lines [34]. Bortezomib may overcome MGMT-related resistance of GBM cell lines to temozolomide [35]. Estradiol is a form of estrogen and induces JNK-dependent apoptosis in human GBM and rat glioma cells [36]. Simvastatin was identified by a recent drug screening study using human cell lines [37]. Decitabine can efficiently induce the differentiation and growth inhibition in IDH1 mutant glioma cells [38].

**Table 4:** Examples in our top 5 % drug predictions for GBM

Drug	Traditional indication
Rosiglitazone	Type 2 diabetes
Bortezomib	Multiple myeloma
Estradiol	Symptoms of menopause
Simvastatin	High cholesterol and triglyceride
Decitabine	Myelodysplastic syndrome

## Discussion

In this study, we predict candidate targeted drugs for GBM through combining discoveries on disease genomics and large-scale mouse phenotype data. We currently have not considered the blood-brain barrier (BBB) permeability of the candidate drugs, which is a major challenge for drug discovery for CNS diseases. No readily available BBB permeable drug database can be publicly accessed to enable simple filtering among the candidate GBM drugs. Computational approaches based on decision tree have been developed to identify BBB permeable drugs [39]. It is also possible to modify the drug chemically or pharmaceutically to increase its permeability [40]. In summary, our future work contains further selecting the candidate GBM drugs that can be delivered into the brain.

TCGA recently classified GBM into four types: Proneural, Neural, Classical and Mesenchymal [6, 8]. Each class has distinct genomic profiles. The Classical GBM has increased EGFR expression and lacks TP53 mutations. The Proneural subtype shows alterations of PDGFRA and point mutations in IDH1. The Neural subtype is characterized by expressions of neuron markers. And the Mesenchymal GBM shows deletions of NF1, expression of mesenchymal markers, and high expressions of the TNF super family pathway and NF- *κ*B pathway [8]. Patients of the four types also respond differently to chemo- and/or radiotherapy [8]. In the future, We will predict drugs for each of the four types targeting on their distinct genetic and genomic features towards achieving precision medicine for GBM. We expect specific and different drug predictions across the GBM subtypes.

In addition, human disease phenotypes, disease phenotypic similarities and drug similarities may also contribute to GBM drug repositioning. For the drug-gene interaction database, we currently use the STITCH database, but other sources like Cancer Cell Line Encyclopedia (CCLE) [41] may contain different knowledge. In the future, we will develop algorithms to seamlessly integrate more comprehensive data to further filter strong candidate GBM drugs. We will also test the candidate drugs in biomedical experiments and clinical studies.

## Conclusions

We screened 1348 approved drugs and predicted targeted drugs for GBM through combining disease genomic and mouse phenotype data. Our approach prioritized the approved GBM drugs, and outperformed a recent drug repositioning method in identifying novel GBM drugs. For all positive examples of GBM drugs, we achieved a median rank of 9.2 %, comparing to 45.6 % generated by the earlier approach. In the paired t-test, our approach generated significantly higher ranks for the evaluation drugs than the baseline approach (*p*=3*e*^−4^). We found that many of our top-ranked predictions have been demonstrated effective in inhibiting the growth of human GBM cells. Overall, the results show that our drug repositioning approach has the potential in finding new targeted therapies for GBM.

## Abbreviations

GBM, glioblastoma; TCGA, the cancer genome atlas; FDA, food and drug administration; MGI, mouse genome informatics; RTK, receptor tyrosine kinase; PI3K, phosphoinositide 3-kinase; STITCH, search tool for interactions of chemicals; BBB, blood-brain barrier; CNS, central nervous system; ccle, cancer cell line encyclopedia; MAP, mean average precisio
